# Retraction: Emotional regulation self-efficacy and impulsivity effects on college students' risk-taking behavior: a cross-sectional study

**DOI:** 10.3389/fpsyg.2026.2023217

**Published:** 2026-10-14

**Authors:** Frontiers Editorial Office

The Journal retracts the 18 June 2025 article cited above.

Following publication, concerns were raised regarding statistical anomalies in the reported results. After assessing the raw data and corrected analyses provided by the authors, the Chief Editor concluded that the concerns were valid. Given the extent of the data-processing errors, the findings are no longer valid, and the article has been retracted.

This retraction was approved by the Chief Editors of Frontiers in Psychology and the Chief Executive Editor of Frontiers.

Frontiers would like to thank Mingzheng Liu for contacting the journal regarding the published article.

